# Cardiovascular magnetic resonance findings in a pediatric population with isolated left ventricular non-compaction

**DOI:** 10.1186/1532-429X-14-9

**Published:** 2012-01-31

**Authors:** Sergio Uribe, Lina Cadavid, Tarique Hussain, Rodrigo Parra, Gonzalo Urcelay, Felipe Heusser, Marcelo Andía, Cristian Tejos, Pablo Irarrazaval

**Affiliations:** 1 Radiology Department, Pontificia Universidad Católica de Chile. Marcoleta 367, Hospital Clinico Universidad Catolica, Santiago 8330024, Santiago Chile; 2 Department of Electrical Engineering, Pontificia Universidad Católica de Chile. Av. Vicuña Mackenna 4860, Macul 7820436, Santiago, Chile; 3 Biomedical Imaging Center, Pontificia Universidad Católica de Chile. Av. Vicuña Mackenna 4860, Macul 7820436, Santiago, Chile; 4 Pediatric Cardiology Department, Pontificia Universidad Católica de Chile. Marcoleta 367, Hospital Clinico Universidad Catolica, Santiago 8330024, Santiago Chile; 5 Division of Imaging Sciences, King's College London. The Rayne Institute, 4th Floor Lambeth Wing St. Thomas' Hospital, London SE1 7EH, UK

**Keywords:** isolated left ventricular non-compaction, cardiomyopathy, spongy myocardium, ventricular performance, prominent trabeculations

## Abstract

**Background:**

Isolated Left Ventricular Non-compaction (LVNC) is an uncommon disorder characterized by the presence of increased trabeculations and deep intertrabecular recesses. In adults, it has been found that Ejection Fraction (EF) decreases significantly as non-compaction severity increases. In children however, there are a few data describing the relation between anatomical characteristics of LVNC and ventricular function. We aimed to find correlations between morphological features and ventricular performance in children and young adolescents with LVNC using Cardiovascular Magnetic Resonance (CMR).

**Methods:**

15 children with LVNC (10 males, mean age 9.7 y.o., range 0.6 - 17 y.o.), underwent a CMR scan. Different morphological measures such as the Compacted Myocardial Mass (CMM), Non-Compaction (NC) to the Compaction (C) distance ratio, Compacted Myocardial Area (CMA) and Non-Compacted Myocardial Area (NCMA), distribution of NC, and the assessment of ventricular wall motion abnormalities were performed to investigate correlations with ventricular performance. EF was considered normal over 53%.

**Results:**

The distribution of non-compaction in children was similar to published adult data with a predilection for apical, mid-inferior and mid-lateral segments. Five patients had systolic dysfunction with decreased EF. The number of affected segments was the strongest predictor of systolic dysfunction, all five patients had greater than 9 affected segments. Basal segments were less commonly affected but they were affected only in these five severe cases.

**Conclusion:**

The segmental pattern of involvement of non-compaction in children is similar to that seen in adults. Systolic dysfunction in children is closely related to the number of affected segments.

## Background

Left Ventricular Non-Compaction (LVNC) is morphologically characterized by numerous prominent trabeculations and deep intertrabecular recesses [1]. It is also known as "spongy myocardium" or "persistent embryonic myocardium", and it is probably secondary to an arrest in the normal process of myocardial compaction during fetal life.

LVNC may be present as an isolated finding (isolated LVNC) or in association with other congenital heart or neuromuscular disorders [2,3]. Distribution of left ventricular segments with non-compaction (NC) can vary from one patient to another, but it often involves the apical, mid-inferior, and mid-lateral myocardial segments [4]. The clinical manifestations, including age of onset, are variable. Functional cardiac status may range from absence of symptoms to severe cardiac disability, sometimes leading to heart transplantation or death [5,6]. Clinical findings include evidence of systolic and diastolic dysfunction, systemic embolism, and severe arrhythmias [7]. Because of the prominent trabeculations, subendocardial ischemia may also result from isometric contraction of the endocardium and myocardium within the deep intertrabecular recesses; this may lead to myocardial scaring, left ventricular dysfunction and arrhythmias [8].

Echocardiography is the most commonly used imaging modality for the diagnosis of LVNC. In some cases however, poor acoustic windows could mislead the diagnosis of this disease, resulting in an erroneous label such as dilated or hypertrophic cardiomyopathy [8]. Cardiovascular Magnetic Resonance (CMR) may outperform echocardiography in defining the morphology and extension of myocardial non-compaction [2]. CMR can also provide excellent delineation of the abnormal trabeculations owing to its higher contrast and resolution. This allows a thorough identification as well as quantification of the extent of non-compaction. Furthermore, CMR also has the ability of detecting myocardial viability [9,10], which has been previously described in patients with LVNC [11].

Different echocardiographic and CMR measurements have been proposed to evaluate the degree of non-compaction. For instance, the ratio between non-compacted and compacted myocardium distance (NC/C ratio) [2,4]; the X:Y ratio described by Chin *et al. *[5], where X represents depth of the trabecular recesses and Y represents total free-wall thickness at the peak of the trabeculations; and the NC area on a two chamber view proposed by Yousef *et al. *[12]. The suggestion from studies in adults is that these measures of non-compaction may correlate with a decrease of Ejection Fraction (EF). For instance, it has been reported that EF is significantly decreased as the extent of non-compaction increases [13]. In children however, there are only a few studies reporting the relation between morphological features of LVNC and ventricular performance. Chin *et al. *[5] found that the echocardiographic ratio X to Y was greater in patients with LVNC compared with controls but this study did not relate this ratio to cardiac function. Pignatelli *et al. *[14] reported that a significant number of children with LVNC could have a deteriorated ventricular function window, followed by a period of transient recovery, and finally a later deterioration stage. Another study performed in children and adults [11], found that in some patients, late gadolinium enhancement (LGE) can be observed not only in non compacted segments but also in normal segments of the heart.

In this study we propose to use CMR to find if there is any correlation between ventricular performance with different morphological features, such as the Compacted Myocardial Mass (CMM), Compacted and Non-Compacted Myocardial Areas (CMA, NCMA), NC/C ratio, distribution of LVNC, and regional wall motion abnormalities in children and young adolescents with isolated LVNC.

## Methods

### Study Population

Institutional Review Board Approval was obtained. All patients or guardians gave permission to use the images for any research purpose when the MR scan was carried out. A retrospective analysis was performed on the CMR images of a pediatric population of 15 children and young adolescents (10 males, mean age 9.7 year old, range 0.6 - 17 year old, SD = 5.2). They were referred to our Radiology Department, between 2005 and 2008, for performance of a CMR scan for confirmation of LVNC. All of them had been previously evaluated with echocardiography and all patients met both echocardiographic and MRI criteria for LVNC. Symptoms such as shortness of breath, dyspnea, palpitations, chest pain, exercise intolerance, and presence of arrhythmias were also recorded.

In six cases, MR imaging was performed under general anesthesia. This procedure was done using a DRÄGER Titus anaesthesia device with pressure-controlled ventilation (routine protocol for young or uncooperative children at our institution). After gas induction, general anesthesia was continued with a continuous i.v. infusion of Remifentanil. All anesthetic procedures were completed without any adverse effects.

### Diagnostic criteria

For the diagnosis of LVNC, we used previously defined diagnostic criteria during the whole study [2]. These were as follows: 1) absence of coexisting cardiac anomalies; 2) presence of multiple prominent trabeculations; 3) presence of multiple deep inter-trabecular recesses and 4) NC/C distance ratio > 2.3, i.e. the maximal end-diastolic distance of the NC endocardial layer to the compaction myocardium layer greater than 2.3.

For this study we considered normal EF values > 53% and indexed CMM values that were between percentiles 25 and 75 [15] or within the confidence interval of normal values [16].

### CMR Protocol

CMR was performed on a Siemens Avanto 1.5T MR system (Siemens, Erlangen, Germany). The protocol included several MR imaging sequences; however, as described in the next section, we only used some of these sequences for the purpose of this study. In particular, the MR protocol included b-SSFP cine images in the horizontal long axis (4-chamber (4Ch) view), short axis (SA), vertical Long Axis (LA) views for ventricular wall motion analysis. The SA view included 10-14 slices covering both ventricles for the assessment of cardiac function. In all patients, a Magnetic Resonance Angiography (MRA) sequence was obtained immediately after injection of a gadolinium-based contrast agent (0.2 mmol/kg of gadopentetate dimeglumine). The same protocol was applied in all patients with the exception of the LGE sequence. From 2007, our Institution began to perform myocardial viability; therefore LGE was performed in only seven patients. LGE consisted of an inversion recovery sequence acquired between 10 to 12 minutes after the MRA sequence. The inversion time was chosen from a Look-Locker sequence [17] selecting the time when blood was nulled. All LGE scans were acquired during mid diastole, the triggering time and acquisition window were set for each patient accordingly to their heart rate. Additionally, a through-plane phase contrast sequence at the level of the Atrio-Ventricular (AV) valves were acquired for flow quantification. The flow scans were acquired with high temporal resolution (between 20 ms and 40 ms depending on the heart rate) to avoid any summation of the e and a waves. A summary of the acquisition parameters for these sequences are depicted in table 1.

**Table 1:** Imaging Parameters of CMR scans

Parameter	Cine b-SSFP	MRA	Flow	LGE
FOV (FH-AP-RL) (mm)	390 × 255	280 × 240 × 140	300 × 270	390 × 255
Acquired Resolution (mm)	2 × 2	2 × 2 × 2	2.1 × 2.1	1.5 × 1.5
Reconstructed Resolution (mm)	1.6 × 1.6	1 × 1 × 1	1.15 × 1.15	1.5
Slice thickness (mm)	8	2	10	7
Nr of slices	1	110 - 180	1	3
TR/TE (ms)	2.9/1.5	3.8/1.9	4.7/2.6	3.2/1.6
TFE factor	9 - 13	15-20	3 - 4	10
Grappa	2 AP	2 AP 1-1.5 RL	no	no
flip angle	60	60	15	15
Nr cardiac phases	20-30	2	20-40	1
Temporal Resolution (ms)	30	60	28.2	70
Triggering modality	Retrospective	none	Retrospective	Gate
Averages	1	1	3	1
V_enc _(cm/s)	NA	NA	150-300	NA

LGE late gadolinium enhancement FH feet head				
AP anterior posterior				
RL right left				

### CMR measurements

In all patients systolic ventricular function was evaluated by measuring the End-Systolic Volume (ESV) and End-Diastolic Volume (EDV). Subsequently, stroke volume and EF were obtained from the last two measurements.

In order to analyze if there was any correlation between ventricular performance and morphological features, different measures including CMM, CMA and NCMA, NC/C ratio, X:Y ratio, distribution of left ventricular non-compaction, and assessment of ventricular wall motion abnormalities were evaluated.

All these morphological and functional measurements were obtained by manual segmentation using commercially available software (Argus, Siemens healthcare). EDV, ESV, CMM, were obtained by manual segmentation of the ventricle and mass in the SA view. For calculating CMA and NCMA, manual segmentation was performed at the end-diastolic phase using LA and 4CH views. Previous studies measured the area of LVNC on the LA view [12]; however, this view only includes the superior and inferior walls. Since non-compaction can also affect the septum and lateral walls, we calculated the total CMA and NCMA as the sum of the areas measured on the LA and 4CH views. All measurements of volumes and areas were indexed to body surface area.

The NC/C distance ratio was measured in the SA or in the 4CH view at the end-diastolic phase of the cardiac cycle for optimal visualization of the 2 layers as proposed in [13].

To measure the distribution of non-compaction, a segmental analysis was evaluated using a standard 17-segment cardiac model as defined by the American Heart Association/American College of Cardiology (AHA/ACC) for standardized myocardial segmentation [18]. As previously recommended, the apex (segment 17) was excluded from the quantitative analysis [2]. Two blinded observers (L.C and S.U.) determined visually in the SA, 4CH and LA views if any particular segment was affected by non compaction (reduced myocardial mass and trabeculations).

Left ventricular wall motion abnormalities were assessed by visual inspection on the SA, 4CH and LA views according to a five point score system as follows: 1: normal, 2: mild to moderate hypokinesia, 3: severe hypokinesia, 4: akinesia, and 5: dyskinesia.

Measurements of the velocity of early to late diastolic filling (E/A) were performed on flow images at the AV valves to find any suggestion of diastolic dysfunction. E/A reversal was recorded as an indication of diastolic dysfunction.

Linear regressions and correlations were calculated for all the different morphological measurements versus ESV, EDV and EF.

## Results

Clinical information included a summary of past medical history. No patient had a family history of any cardiomyopathy. The most common clinical findings were arrhythmias, present in five patients, with two patients having the Wolff-Parkinson-White syndrome. Three patients complained of dyspnea, two of chest pain and four patients of palpitations. Only one patient was asymptomatic.

Table 2 and 3 summarizes the functional and morphological CMR findings. The mean EF was 51.7% (SD = 10.93%, and ranging from 28.4% to 64.2%) and only five patients had a decreased EF (< 53%). The E/A ratio suggested that four of them had diastolic dysfunction as well. There was only one patient with diastolic dysfunction but normal EF. Another five patients had abnormal CMM values (greater than percentile 97 according to [15]), but none of them had decreased EF. LGE was not present in patients (n = 7) who underwent the LGE sequence.

**Table 2:** Summary of the analyzed functional and morphological CMR parameters.

	EF (%)	EDV (cc/m^2^)	ESV (cc/m^2^)	CMM (g/m^2^)	CMA (cm^2^/m^2^)	NCMA (cm^2^/m2)	NC/C distance ratio
**Mean/SD**	51.69/10.93	83.99/14.94	40.67/13.17	61.47/16.79	21.68/6.46	25.47/8.33	3,83/0,79
**Range**	28.40- 64.20	60.10-111.80	27.40- 80.00	44.50-105.40	15.95 - 40.22	15.91-49.09	2.57 -5.05

EF, ejection Fraction

EDV, end-diastolic Volume

ESV, end-systolic volume

CMM, compacted myocardial mass

CMA, compacted myocardial Area

NCMA, non compacted myocardial area.

NC/C, non-compaction divided by compaction

**Table 3:** Description of CMR morphological and cardiac function findings in children and young adolescent with isolated LVNC.

Patient	Age y.o	EF %	EF < 50%	Abnormal CMM	Diastolic Dysfunction	LGE	NC/C ratio	Wall Motion Abnormalities	Numbers of LV segments with NC
1	10.4	58	No	Yes	No	N/A	4,7	Nono	6
2	14.1	64.2	No	No	No	N/A	4,6	No	3
3	3.4	**44.6**	**Yes**	**No**	**Yes**	N/A	5	Yes	**10 (NC in basal segment)**
4	17	53.5	No	Yes	No	N/A	2,9	No	6
5	14.1	53.3	No	No	No	Negative	3,7	No	4
6	8.9	61.6	No	Yes	No	Negative	3,7	No	4
7	14.1	54.4	No	No	No	N/A	3,7	No	6
8	11.5	63.4	No	No	No	Negative	3,7	No	6
9	0.6	**28.4**	**Yes**	**No**	**Yes**	Negative	4,7	Yes	**10 (NC in basal segment)**
10	4.3	**35.7**	**Yes**	**No**	**Yes**	N/A	5	Yes	**11 (NC in basal segment)**
11	13.1	60.5	No	Yes	No	Negative	3	No	4
12	11.2	53.6	No	No	Yes	N/A	2,6	No	4
13	1.9	**41.5**	**Yes**	**No**	**Yes**	N/A	3,6	Yes	**12 (NC in basal segment)**
14	5.5	**41.5**	**Yes**	**No**	**No**	Negative	3,6	Yes	**11 (NC in basal segment)**
15	14.8	61.2	No	No	No	Negative	3	Yes	6

CMM, compacted myocardial Mass

EF, ejection Fraction

LGE, Late gadolinium enhancement

NC/C, non-compaction divided by compaction

LV, left ventricular

N/A, not applicable

**Figure 1:**
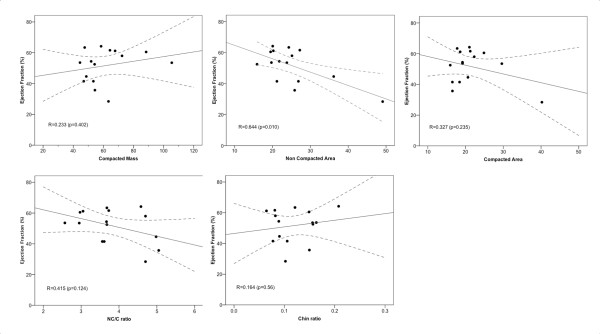
**Linear regression and correlations between EF and different morphological measurements**. There was a low correlation between all these parameters and EF. None of these measurements are a good indicator of ventricular performance in children with isolated LVNC. (Compacted mass is in g/m^2 ^and measures of areas are in cm^2^/m^2^).

**Figure 2:**
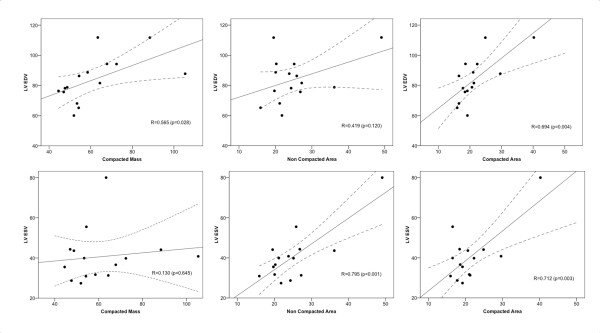
**Linear regression and correlations**. Linear regression and correlations between myocardial compacted mass, non compacted area, and compacted area with ESV and EDV were higher than those found in for EF (Figure 1). However, linear regression plots were also scattered indicating a poor linear relationship between these morphological indexes and ESV or EDV. (LV-ESV and LV-EDV are in cc/m^2^, compacted mass in g/m^2 ^and measures of area are in cm^2^/m^2^).

**Figure 3:**
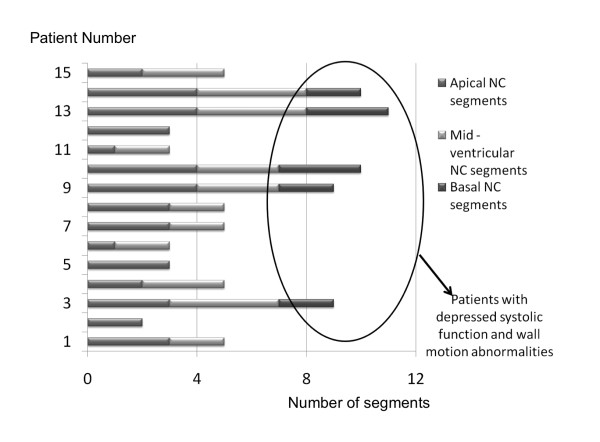
**Distribution of segments with LVNC in all patients**. Those patients with more than 9 affected segments were the only ones with compromise of left ventricular performance. Basal segment involvement tended to occur in more severe cases.

**Figure 4:**
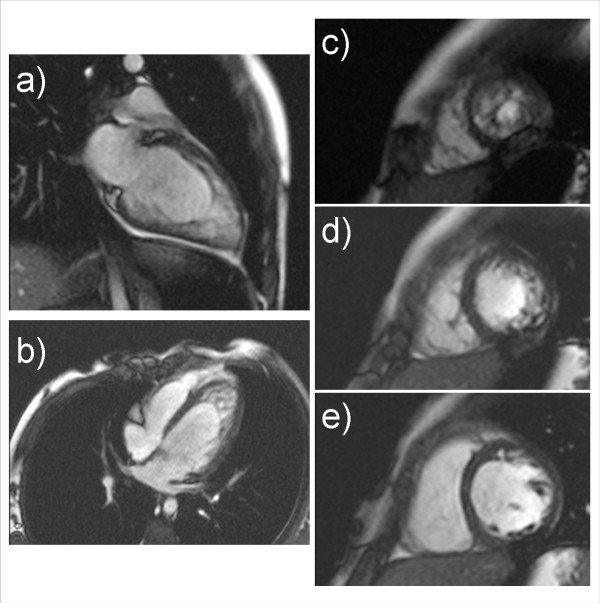
**A five years old female with EF 41.5%**. Steady-state free precession magnetic resonance imaging in a Long Axis (a), four-chamber (b) and short-axis (c, d, e) views demonstrate extensive trabeculations of the LV wall in the basal (e), mid (d) and apical segments (c).

Figure 1 shows the linear regression and correlations between EF and the five morphologic measurements: NC/C ratio, CMM, CMA, NCMA and the X:Y ratio. As shown in this figure, the highest correlation was 0.64 for the relation between NCMA and EF. Although the mean NC/C ratio was relatively high (3.82 ranging from 2.57 to 5.05) and the X:Y ratio indicated the presence of LVNC, there was a poor correlation between both measurements and EF. Better correlations were obtained when comparing CMM, CMA, and NCMA with ESV and EDV (Figure 2). However, based on the linear regression plots it is difficult to establish linear relationships between any of these measurements. Distribution of segments with LVNC morphology is shown in table 4 and Figure 3. Non-compaction of the left ventricle was more common in the apical level, present in all patients (100%), as compared with the mid-ventricular level (80%) and the basal level (33%). In 26,6% of the patients the non-compaction involved all four segments at the apical level. At the mid-ventricular level, inferior and lateral segments were more commonly affected. No patients had non-compaction in the septal wall at the mid-ventricular or basal level. The mean number of affected segments was 5.86 (range: 2-11). We found five patients with ≥ 9 segments affected with non-compaction. These patients were the only ones with non-compaction also at the basal ventricular level (Figure 3). Figure 4 shows the images obtained in one patient with severe isolated LVNC.

**Table 4:** Distribution of non-compaction in different segments at different levels of the left ventricle.

	Basal	Med	Apical	Total
Anterior	2	6	13	**21**
Inferior	2	4	10	**16**
Septal	NA	NA	4	**4**
Lateral	NA	NA	15	**15**
Anteroseptal	0	0	NA	**0**
Inferoseptal	0	0	NA	**0**
Inferolateral	4	12	NA	**16**
Anterolateral	4	12	NA	**16**

**Total**	**12**	**34**	**42**	**88**

**Figure 5:**
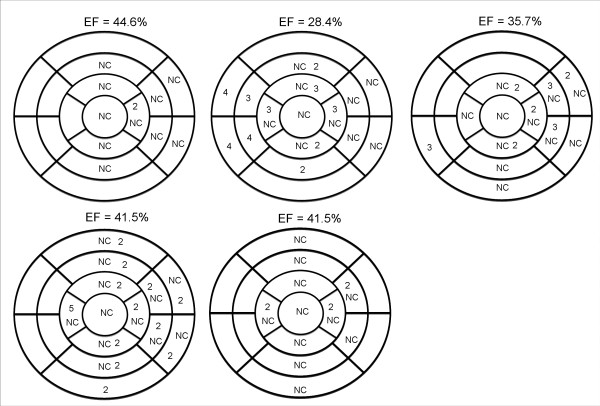
**Result of the 17-segment analysis of wall motion abnormality and non compation (numbers indicate degree of wall motion abnormalities according to text)**. These patient were the only ones who had wall motion abnormalities and and Ejection Fraction (EF) < 53%. Interestingly, in the more severe cases there were wall motion abnormalities in septal segments non-affected by no compaction.

Only five patients had wall motion abnormalities (Figure 5). Interestingly, this figure shows that in the more severe cases, wall motion abnormalities were seen in some segments not affected with non-compaction, involving especially some septal segments at the apex, mid and basal levels. As can be seen in Table 4 and Figure 3, those five patients with wall motion abnormalities were the only ones with a reduced EF < 53%, with more than nine segments affected with non-compaction and four of them had evidence of left ventricular diastolic dysfunction.

## Discussion

Several echocardiographic and CMR measurements have been proposed to diagnose LVNC. There has been an association between adult patients with isolated LVNC and depressed left ventricular function; however the reason for this remains unclear. In adults, it has been found that some of these measurements such as NC/C ratio and NCMA are well correlated with systolic dysfunction. However, in children these relations have not been yet established.

In this study we investigated the relation between different anatomical measurements with ventricular performance using CMR on a pediatric population. We found that only five patients had a decreased EF (< 53%) and that there was a poor correlation between EF and measurements such as CMM, CMA, NC/C ratio and the X:Y ratio proposed by Chin *et al. *[5]. A better correlation was found between EF and NCMA (R = 0.67). Yousef *et al. *[12] found a similar correlation but in an adult population, with the NCMA being measured in a LA view. In our study the NCMA was calculated as the sum of the NCMA on LA and 4Ch views; this was done to include any septal and lateral walls that may involve any degree of non-compaction.

We also found that the relation between the evaluated morphological indices with ventricular dysfunction was poor in children with LVNC. There was a small association between the degree of non-compaction and ventricular dysfunction; however it was difficult to establish a linear relationship between these parameters. This is probably explained by the greater cardiac reserve present in a younger population. However, it is important to continue assessing ventricular function in these patients along time, since it has been shown that this variable could potentially deteriorate with age. All these observations suggest that ventricular dysfunction becomes more overt with increasing age [14,19,20]. Recently, Pignatelli *et al. *[14] reported that nine patients presenting depressed LV contractility in the first year of life and then had a transient recovery of LV function; however, LV EF deteriorated later in life.

In our study, the severity and extension of non-compaction was quantified by calculating the number and distribution of 16 myocardial segments that had some degree of non-compaction. According to the literature, in adults, the distribution of left ventricular non-compaction is mainly at the apical (all segments), mid-inferior and mid-lateral segments [4]. In our pediatric population, the distribution of segments affected by non-compaction was very similar to that found in adults.

Additionally, we found that if any basal segment showed some degree of non-compaction, regional ventricular contraction was abnormal causing a depression of systolic and/or diastolic function. In particular, we found that five patients had nine or more segments with ventricular non-compaction, and all of them had at least one basal segment with non-compaction and presented regional wall motion abnormality. Importantly, the same patients were the only ones with a decreased EF and four also had diastolic dysfunction. Other studies performed in adults, have shown a similar relation between the extension of non-compaction and the severity of this disease [12,13]. Dodd et al [13], showed that the extension of left ventricular non-compaction at the mid ventricular levels or the presence of LGE patterns at basal levels, imply more severe clinical disease in an adult population. These studies are on line with our findings, which indicate that the severity of the disease in children may be determined when non-compaction extend to the basal segments.

LGE has been previously detected using CMR in patients with LVNC. However, our results from CMR late gadolinium enhancement were negative for all patients. Future studies may consider using stress perfusion with CMR. This technique could potentially provide valuable information about microcirculation in the myocardium and performance of the heart [13,20-22].

One drawback of the study is the limited clinical information available from patients. This information was difficult to obtain since most were outpatients referred from other institutions. We are currently planning to perform a follow up study of these patients to analyze any possible relation between clinical symptoms and image findings. Another limitation is that the study was a retrospective review of a relatively small number of patients. However, to the best of our knowledge, this is the largest series of children and young adolescents with isolated LVNC assessed with CMR. Even though, we performed a retrospective review of the CMR images, the same CMR protocol, (with the exception of LGE), was applied in all patients, providing consistent data for assessment of morphology and cardiac function parameters.

Although echocardiography provides good acoustic windows in children, in pediatric patients with LVNC, CMR may outperform echocardiography. MR allows a comprehensive evaluation of the heart including: measuring accurately cardiac function, cardiac volumes, CMM, NCMM; and visualizing and analyzing all segments of the ventricles in greater detail which may be difficult using echocardiography. Furthermore, MR allows us performing myocardial viability that is not possible with echocardiography.

## Conclusions

In conclusion, in children and young adolescents with isolated LVNC, none of the anatomical measurements including CMM, CMA, NC/C ratio and the X:Y ratio had a good correlation with cardiac function, only NCMA had a better relation with EF but this was still low. Nevertheless, the number of affected segments was a strong predictor of ventricular function. The segmental pattern of involvement in childhood was similar to that found in previous adult studies. Furthermore, involvement of basal segments in our cohort was confined to those with more extensive disease.

## Competing interests

The authors declare that they have no competing interests.

## Authors' contributions

SU conceive the study and participated in its design, coordination, collection and statistical analysis of the data, and drafting of the manuscript.

LC participated in the design of the study, acquisition and analysis of the data, and drafting the manuscript.

GU participated in the design of the study and drafting the manuscript.

TH participated in analysis of the data, and drafting the manuscript.

RP participated in the acquisition and analysis of the data, and drafting the manuscript.

FH participated in the design of the study and drafting the manuscript.

MA participated in the statistical analysis of the data, and drafting the manuscript.

CT participated in the design the study, and drafting of the manuscript

PI participated in the design the study, and drafting of the manuscript

All authors read and approved the final manuscript.
